
JOURNAL INFORMATION
==============================
NLM Title Abbreviation: ScientificWorldJournal
Journal ID: ScientificWorldJournal
NLM Journal ID: 101131163
Journal ID: TSWJ

The Scientific World Journal

ISSN: 2356-6140
EISSN: 1537-744X
Publisher: Wiley

ARTICLE INFORMATION
==============================
PMCID: PMC6084117
PMID: 30147494
DOI: 10.1100/tsw.2001.151
Article ID: 860682
Article version: 1
Subjects: Short Report

Differential Processing of Cytosolic and Mitochondrial Caspases

Yuan Hua
Mutomba Martha
Heiser Marc
Gottlieb Roberta A. *
Dept. of Molec. and Exp. Medicine Scripps Res. Inst., 10550 N. T., San Diego, CA, USA
*Roberta A. Gottlieb: robbieg@scripps.edu
Publication date: 2001
Electronic publication date: 2001 Nov 21
Volume: 1
First page: 42
Last page: 42
Copyright: Copyright © 2001 Hua Yuan et al.
Copyright year: 2001
License: This is an open access article distributed under the Creative Commons Attribution License, which permits unrestricted use, distribution, and reproduction in any medium, provided the original work is properly cited.
License URL: https://creativecommons.org/licenses/by/3.0/

Funding: National Institutes of Health R01 AG13501 National Institutes of Health R01 HL61518

==============================
Miami *Nature Biotechnology* Short Reports
*TheScientificWorld* (2001) 1(S3), 42SR
ISSN 1532-2246; DOI 10.1100/tsw.2001. 151

### DIFFERENTIAL PROCESSING OF CYTOSOLIC AND MITOCHONDRIAL CASPASES

Hua Yuan, Martha Mutomba, Marc Heiser, and Roberta A. Gottlieb*

Department of Molecular & Experimental Medicine, The Scripps Research Institute, 10550 N. Torrey Pines Rd, La Jolla, CA 92037, USA
* robbieg@scripps.edu

**INTRODUCTION.** The mitochondria have been shown to play an important role in the initiation of caspase activation during apoptosis. Recently, some caspases have been shown to be associated with mitochondria (1,2,3). In this report we show the submitochondrial localization of caspases-3, and -9, and the conditions under which mitochondrial caspase activation may occur, as well as the effect of Bcl-2 on caspase processing.

**METHODS.** In Jurkat, CEM/Neo and CEM/Bcl-2 cell lines, apoptosis was induced with anti-Fas antibody (clone CH-11, Kamiya Biomedical Co.) or staurosporine (Sigma). Mitochondria were prepared from cells after disruption by nitrogen cavitation (4).

**RESULTS.** Our studies indicated that caspases-3 and -9 were associated with the washed mitochondrial pellet as well as in the cytosol. Caspase-3 was present in the inter membrane space while caspase-9 was located both on the outside of the mitochondrial outer membrane as well as in the intermembrane space. During receptor-mediated apoptosis, caspase-3 processing was much more extensive in the cytosolic fraction than in mitochondrial fraction 1 hour after Fas ligation. However, staurosporine treatment resulted in caspase-3 cleavage observed at 4 hours in both cytosol and mitochondria and with approximately similar kinetics. Caspase-9 processing in mitochondria indicated that the cytochrome c-dependent 35 kDa and caspase-3-dependent 37 kDa forms were approximately equal in abundance while the 37 kDa form predominated in cytosol. Interestingly, the amount of processed caspase-9 increased in the mitochondrial fraction over time in response to both stimuli. Bcl-2 largely suppressed caspase processing in both cytosolic and mitochondrial fractions.

**DISCUSSION.** We have demonstrated that caspases-3 and -9 are associated with mitochondria in Jurkat cells. The subcellular distribution of caspases may vary from cell type to cell type as suggested by Krajewski (2). The enrichment of processed caspase-9 in the mitochondrial fraction may suggest that the apoptosomes transiently nucleate on mitochondria and then relocate to the cytosol. Mitochondrial and cytosolic caspases are activated differentially in response to Fas ligation and staurosporine treatment. During staurosporine-induced apoptosis, mitochondria may function as a trigger for caspase activation and rapidly lead to the processing of caspases in the cytosolic compartment. Bcl-2 overexpression suppressed cytochrome *c* release and largely prevented caspase processing in both cytosol and mitochondria in response to Fas ligation and staurosporine treatment. The significance of a pool of mitochondrial caspases is unclear, unless they are there to proteolyze mitochondrial proteins. More work is needed to determine the functional significance of these mitochondrial caspases as well as to determine the mechanisms regulating their activity.

**ACKNOWLEDGEMENTS.** This work was supported by NIH R01 AG13501 and R01 HL61518 (to R.A.G.).

**REFERENCES.**

1. Mancini, M., Nicholson, D.W., Roy, S., Thornberry, N.A., Peterson, E.P., Casciola-Rosen, L.A., and Rosen, A. (1998) J. Cell Biol. 140, 1485-1495
2. Krajewski, S., Krajewska, M., Ellerby, L.M., Welsh, K., Xie, Z., Deveraux, Q.L., Salvesen, G.S., Bredesen, D.E., Rosenthal, R.E., Fiskum, G., and Reed, J.C. (1999) Proc. Natl. Acad. Sci. U S A 96, 5752-5757
3. Susin, S.A., Lorenzo, H.K., Zamzami, N., Marzo, I., Brenner, C., Larochette, N., Prevost, M.C., Alzari, P.M., and Kroemer, G. (1999) J. Exp. Med. 189, 381-394
4. Adachi, S., Gottlieb, R.A., and Babior, B.M. (1998) J. Biol. Chem. 273, 19892-19894
